# Complete Genome Sequences of Five Salmonella enterica Strains Used in Inoculation Cocktails in Low-Moisture Food Storage Studies

**DOI:** 10.1128/MRA.01588-18

**Published:** 2019-01-10

**Authors:** Julie Haendiges, Susanne Keller, Quincy Suehr, Nathan Anderson, Elizabeth Reed, Jie Zheng, Jesse D. Miller, Maria Hoffmann

**Affiliations:** a NSF International, Applied Research Center, Ann Arbor, Michigan, USA; b Center for Food Safety and Applied Nutrition, Division of Microbiology, Office of Regulatory Science, U.S. Food and Drug Administration, College Park, Maryland, USA; c Center for Food Safety and Applied Nutrition, Division of Food Processing Science and Technology, Office of Food Safety, U.S. Food and Drug Administration, Bedford Park, Illinois, USA; Loyola University Chicago

## Abstract

Survival kinetics of Salmonella enterica have been previously studied using an inoculum cocktail composed of different strains that have been associated with low-moisture foods. Here, we report the closed genome sequences of five strains of Salmonella enterica that are commonly used in these storage studies.

## ANNOUNCEMENT

The incidence of salmonellosis associated with dry food products has been increasing in recent years. An aspect of public health concern is the ability of Salmonella spp. to survive on these foods (nuts, black pepper, and spices) in a low-moisture environment. Numerous studies on the survival of Salmonella enterica in dried foods have previously been published (1–3) using a cocktail of different strains. Here, we report the complete closed genome sequences of 5 strains commonly used in these studies, S. enterica serotype Anatum CFSAN076215 (strain 6802), isolated from peanuts; S. enterica serotype Enteritidis CFSAN076214 (strain ATCC BAA-1045), isolated from almonds; S. enterica serotype Oranienburg CFSAN076211 (strain 1839), isolated from pecans; S. enterica serotype Tennessee CFSAN076210 (strain K4643), isolated from peanut butter; and S. enterica serotype Mbandaka CFSAN076213 (strain 688538), isolated from tahini.

The isolates were cultured in Trypticase soy broth (Becton, Dickinson, Franklin Lakes, NJ, USA) overnight at 37°C. The genomic DNA was isolated using the DNeasy blood and tissue kit (Qiagen, Inc., Valencia, CA, USA). A single SMRTbell 20-kb library was prepared according to the 20-kb PacBio sample preparation protocol using the BluePippin size-selection system (Sage Science, Beverly, MA, USA). Each isolate was sequenced based on previously reported procedures on the PacBio RS II platform (Pacific Biosciences, Menlo Park, CA, USA) using a single small-molecule real-time (SMRT) cell (4). The sequencing statistics for each isolate can be found in Table 1. The genomes were *de novo* assembled using the Hierarchical Genome Assembly Process version 3.0 using default settings. The assembled sequences were annotated using the NCBI Prokaryotic Genomes Annotation Pipeline (PGAP) and have been deposited at DDBJ/EMBL/GenBank. The S. Anatum (CFSAN076215) chromosome size was 4,689,440 bp, with a G+C content of 52.1%, and the plasmid size was 104,123 bp, with 50.5% G+C content. The plasmid (pCFSAN076215) showed two toxin-antitoxin (TA) modules (*ccdA-ccdB* and *higA-2–higB-2*). The *ccdA-ccdB* TA module can lead to the formation of persister cells if induced by environmental stress. (5). The S. Oranienburg (CFSAN076211) genome size was 4,651,134 bp, with 52.1% G+C content. The S. Tennessee (CFSAN076210) chromosome size was 4,834,056 bp, with 52.2% G+C content, and the plasmid was 109,917 bp, with 50.7% G+C content. The plasmid (pCFSAN076210) has the oxidative stress gene *grxA*, which can be upregulated in response to preadaptation to cold stress and may provide increased protection against hydrogen peroxide (6). The S. Enteritidis (CFSAN076214) chromosome size was 4,668,874 bp, with 52.0% G+C content; plasmid 1 was 59,261 bp, with 42.1% G+C content, and plasmid 2 was 56,636 bp, with 51.6% G+C content. Plasmid 1 (pCFSAN076214_1) has *vir* genes associated with the type IV secretion system. Plasmid 2 (pCFSAN076214_2) has virulence genes *spvA, spvB, spvC, spvD,* and *spvR*, which have been shown to express lethal disease in BALB/c mice (7). The S. Mbandaka (CFSAN076213) genome size was 4,709,669 bp, with 52.3% G+C content.

**TABLE 1:** PacBio RS sequencing statistics

Isolate name	Coverage (×)	Total no. of reads	Avg read length (bp)	*N*_50_ read length (bp)
CFSAN076210	169	90,179	12,732	16,680
CFSAN076211	115	62,043	11,025	20,045
CFSAN076213	120	69,380	10,357	18,170
CFSAN076214	162	77,741	12,017	16,680
CFSAN076215	182	77,545	13,499	26,714

### Data availability.

The sequences have been deposited in GenBank under the following accession numbers (SRA accession numbers): CP033338 and CP033339 (SRR8217764) for S. Anatum, CP033344 (SRR8170031) for S. Oranienburg, CP033345 and CP033346 (SRR8170030) for S. Tennessee, CP033340, CP033341, and CP033342 (SRR8170032) for S. Enteritidis, and CP033343 (SRR8170034) for S. Mbandaka.

## References

[1] Zhang Y, Keller SE, Grasso-Kelley EM 2017 Fate of *Salmonella* throughout production and refrigerated storage of tahini. J Food Prot 80:940–946. doi:10.4315/0362-028X.JFP-16-507. 28463084

[2] Keller SE, Anderson NM, Wang C, Burbick SJ, Hildebrandt IM, Gonsalves LJ, Suehr QJ, Farakos SMS 2018 Survival of *Salmonella* during production of partially sprouted pumpkin, sunflower, and chia seeds dried for direct consumption. J Food Prot 81:520–527. doi:10.4315/0362-028X.JFP-17-318. 29513105

[3] Farakos SMS, Pouillot R, Keller S 2017 *Salmonella* survival kinetics on pecans, hazelnuts, and pine nuts at various water activities and temperatures. J Food Prot 80:879–885. doi:10.4315/0362-028X.JFP-16-392. 28414256

[4] Hoffmann M, Payne J, Roberts RJ, Allard MW, Brown EW, Pettengill JR 2015 Complete genome sequence of Salmonella enterica subsp. enterica serovar Agona 46004 2-1, associated with a multistate outbreak in the United States. Genome Announc 3:e00690-15. doi:10.1128/genomeA.00690-15. 26139714 PMC4490842

[5] Tripathi A, Dewan PC, Barua B, Varadarajan R 2012 Additional role for the ccd operon of F-plasmid as a transmissible persistence factor. Proc Natl Acad Sci USA 109:12497–12502. doi:10.1073/pnas.1121217109. 22802647 PMC3412025

[6] Shah J, Desai PT, Chen D, Stevens JR, Weimer BC 2013 Preadaptation to cold stress in Salmonella enterica serovars Typhimurium increases survival during subsequence acid stress exposure. Appl Environ Microbiol 79:7281–7289. doi:10.1128/AEM.02621-13. 24056458 PMC3837722

[7] Karuse M, Roudier C, Fierer J, Harwood J, Guiney D 1991 Molecular analysis of the virulence locus of the Salmonella Dublin plasmid pSDL2. Mol Microbiol 5:307–316. doi:10.1111/j.1365-2958.1991.tb02111.x. 2041471

